# Indigenous traumatic brain injury research: responding to recruitment challenges in the hospital environment

**DOI:** 10.1186/s12874-019-0813-x

**Published:** 2019-08-07

**Authors:** Michelle S. Fitts, Taeha Condon, John Gilroy, Katrina Bird, Erica Bleakley, Lauren Matheson, Jennifer Fleming, Alan R. Clough, Adrian Esterman, Paul Maruff, India Bohanna

**Affiliations:** 1 College of Public Health, Medical & Veterinary Sciences, Division of Tropical Health and Medicine, James Cook University, Townsville, QLD Australia; 2 Centre for Alcohol Policy Research, La Trobe University, Melbourne, VIC Australia; 3 College of Public Health, Medical & Veterinary Sciences, Division of Tropical Health and Medicine, James Cook University, Cairns, QLD Australia; 4 Faculty of Health Sciences, Centre for Disability Research and Policy, The University of Sydney, Sydney, Australia; 5 National Critical Care and Trauma Response Centre, Royal Darwin Hospital, Darwin, Northern Territory Australia; 6 Occupational Therapy Department, Townsville Hospital and Health Service, Townsville, QLD Australia; 7 School of Health and Rehabilitation Sciences, The University of Queensland, Brisbane, Australia; 8 Community-based Health Promotion and Prevention Studies Group, Australian Institute of Tropical Health and Medicine, James Cook University, Cairns, QLD Australia; 9 Sansom Institute for Health Research and School of Nursing and Midwifery, University of South Australia and Australian Institute of Tropical Health and Medicine, James Cook University, Cairns, QLD 4870 Australia; 10 Florey Institute of Neuroscience and Mental Health, Melbourne, Australia

**Keywords:** Methodology, Recruitment, Brain injury, Aboriginal and Torres Strait islander health, Disability, Westmead post-traumatic amnesia scale

## Abstract

**Background:**

Hospitals are common recruitment sites for injury and disability studies. However, the clinical and rehabilitation environment can create unique challenges for researchers to recruit participant populations. While there is growing injury and disability focused research involving Indigenous people to understand the types of services and supports required by this population to enhance their recovery experiences, there is limited knowledge of researchers’ experiences implementing recruitment processes in the tertiary hospital environment. This paper reflects on the specific challenges of recruiting Indigenous patients following a traumatic brain injury from two tertiary hospitals in Northern Australia.

**Methods:**

Between July 2016 and April 2018, research staff recruited eligible patients from one hospital in Queensland and one hospital in the Northern Territory. Qualitative records summarising research staff contact with patients, family members and clinical hospital staff were documented. These qualitative records, in addition to field trip notes and researcher reflections were reviewed to summarise the main challenges in gaining access to patients who fit the eligibility criteria.

**Results:**

During the recruitment process, there were five main challenges encountered: (1) Patients discharging against medical advice from hospital; (2) Discharge prior to formal emergence from Post Traumatic Amnesia as per the Westmead Post Trauma Amnesia Scale; (3) Patients under adult guardianship orders; (4) Narrow participant eligibility criteria and (5) Coordinating around patient commitments and treatment. Details of how the recruitment processes were modified throughout the recruitment phase of the study to ensure greater access to patients that met the criteria are described.

**Conclusion:**

Based on our recruitment experiences, several recommendations are proposed for future TBI studies with Indigenous Australians. In addition to treatment, Indigenous TBI patients have wide range of needs that must be addressed while in hospital. Patient engagement and data collection processes should be flexible to respond to patient needs and the hospital environment. Employment of a centralized recruiter at each hospital site may help to minimise the challenges researchers need to navigate in the hospital environment. To improve recruitment processes in hospitals, it is essential for researchers examining other health or injury outcomes to describe their recruitment experiences.

## Background

Traumatic brain injury (TBI) is a major cause of death and disability for Indigenous people living in colonised nations including Australia, New Zealand, Canada and the United States compared to their non-Indigenous counterparts [1–6]. Australia’s First Peoples, Aboriginal and Torres Strait Islander (Indigenous) people, have an incident rate of TBI that is at least twice as high as the non-Indigenous population (166.4 per 100,000 compared to 97.8 per 100,000) [1]. Indigenous Australians that sustain a TBI are more likely to be injured through an assault, to be female, to live remotely and to have comorbidities [2]. Between 1999 and 2005, the rate of head injury including TBI, due to assault among Indigenous Australians, was 854.8 per 100,000 population, or 21 times the non-Indigenous rate (40.7 per 100,000 population) [3]. For Indigenous women, the rate of head injury due to assault was 69 times the rate experienced by non-Indigenous women [3]. In light of the high rates of head injury Indigenous Australians experience [1–3], there is a growing number of studies dedicated to understanding the experiences of Indigenous peoples who have sustained TBI to develop appropriate interventions to support them [7, 8].

Studies investigating the lived experience of people with TBI, particularly the ‘transition period’ from hospital to home play a fundamental role in understanding patients’ hospital experiences, service and support access and health outcomes [9–12]. In clinical and rehabilitation research including TBI, white, middle class individuals tend to be overrepresented and people from socio disadvantaged groups under-represented [13]. Researchers continue to struggle to access, engage and retain participants from socially disadvantaged groups [14]. The acute and rehabilitation environment presents an additional and unique set of challenges for researchers to navigate. Inability to participate due to poor health, queries over patient capacity, timing of recruitment and the length of time required to identify suitable patients are among the barriers affecting participant recruitment in the acute and rehabilitation environment [15, 16]. Although there is a growing body of literature reporting on the barriers to sampling recruitment and retention of socioeconomically disadvantaged groups in health research, and strategies for increasing recruitment [14], there is a lack of literature on recruitment of Indigenous patients from acute and rehabilitation environments. This is likely to impact the ability of researchers to develop appropriate recruitment strategies before research commences. Conversely, if researchers were armed with knowledge regarding effective recruitment strategies, this could have a positive impact by enhancing the effectiveness of TBI research with Indigenous Australians.

The authors have undertaken a National Health and Medical Research Council (NHMRC)-funded project (APP1081947) aiming to understand transition outcomes, including cognitive health, wellbeing and recovery, for Indigenous people during the six-month transition period from hospital to home following a TBI [7]. The original study included two major trauma hospitals in Northern Australia, the Royal Darwin Hospital (RDH), Northern Territory and The Townsville Hospital (TTH), Queensland. Both hospitals service large geographical areas (see Figure, Box 1). Cairns and Mount Isa Hospitals were later added as recruitment sites. This commentary summarises the experiences of the research team in undertaking recruitment for this project and offers recommendations for other researchers conducting similar studies in the acute and rehabilitation environment.

## Methods

### Design

As a longitudinal study [5], information from participants was collected at three time points: prior to discharge from hospital, three months and six months post-discharge. This study and the larger project [5] were underpinned by Indigenous Standpoint Theory [17, 18]. The theory is not an “Indigenous” way of doing research but rather, the theory offers an alternative to the practice of subjugating Indigenous people as the *cultural other* through prioritising the personal experiences of Indigenous peoples in the research process. Indigenous Standpoint Theory ensures the research is planned, owned and controlled by Indigenous people and ensure that Indigenous people are intimately involved with all aspects of the research [19]. Through capturing and reflecting on patients’ experiences, as well as our own as researchers in the hospital, this study demonstrates how Indigenous peoples’ (patients) personal experiences have influenced the protocol and processes of this study.

### Participants

**Fig. 1:**
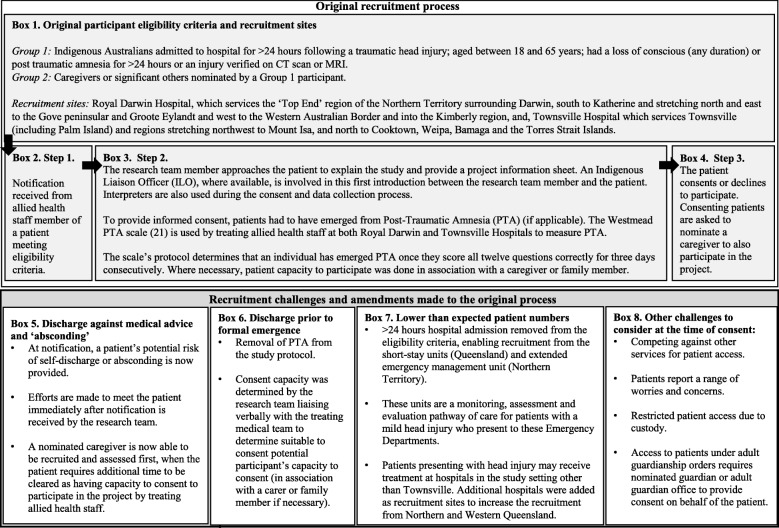
Description of the original recruitment process, recruitment challenges and related amendments

Approximately 200 participants with TBI and their care givers were planned to be recruited. Full recruitment procedure information is available in the study protocol [7]. The recruitment period occurred between July 2016 and February 2018. As presented in Box 1, Fig. 1, two groups of participants were recruited to the project: i) Aboriginal and Torres Strait Islander patients admitted for a TBI and ii) caregivers. Patients were required to meet the following criteria: (1) identify as Aboriginal and Torres Strait Islander; (2) admitted for a TBI (3) aged between 18 and 65 years; (4) be able to provide informed consent; (5) have adequate communication skills to participate. Caregiver inclusion criteria included being aged 18 years or older and able to provide informed consent. Caregiver recruitment to the study was designed to allow the patient with TBI to self-select members for participation who met the inclusion criteria. Nomination of a caregiver was not a requirement of the study and did not preclude patients from participating in the study.

### Recruitment approaches

The original recruitment strategy was a three-step process: Step 1. A nominated allied heath staff member identified an eligible patient and notified the research team; Step 2. Upon notification, a research team member approached the patient to explain the study and provide an information sheet (Box 3); Step 3. Once consent had been obtained and the assessment completed, patients were invited to nominate a caregiver or significant family member to also participate in the study (Box 4).

Patients had to have emerged from post-traumatic amnesia (PTA) and be deemed by a medical professional suitable to be able to provide informed consent. To measure PTA, allied health staff used the Westmead PTA Scale [20]. According to the scale, which consists of memory and orientation-related questions, an individual is deemed to have emerged from PTA once all twelve questions are answered correctly for three consecutive days [21]. All patients who provided informed consent could participate in the study. This included patients who consented in the hospital but discharged against medical advice (DAMA). In this study, ‘capacity to consent’ is defined as the patient’s ability to retain information and understand the requirements and consequences of participation in the project.

Based on the recruitment experiences described here, we will recommend that a carefully-constructed protocol, augmented by regular discussion between research and hospital staff, and subsequent revision of recruitment process are required to respond to working in an acute and rehabilitation environment.

### Research team

During the course of the project there were six project staff who recruited patients and caregivers. The project staff had previously worked in the areas of occupational therapy, social work, psychology and Indigenous public health research. Between them, they held many person-years of experience working with and engaging Aboriginal and Torres Strait Islander people and communities in a range of research projects including follow-up on illicit substance misuse including cannabis and behaviours largely associated with TBI such as unhealthy alcohol use and drink driving. Prior to data collection, the project staff completed relevant training including an accidental counselling short course, and training in Indigenous research methodologies and qualitative inquiry (lead by author JG).

### Documentation of project staff recruitment experiences

During the project, research team members recorded any interaction (face-to-face, telephone, email) with patients, family members of patients and hospital staff in a central project database. The database contained sensitive information and was stored according to university policy. For each interaction, the date and type of interaction, key points interaction, and length of time of interaction were recorded. For more significant interactions, researcher reflections and action points were also documented. First author (MF) reviewed the database and research reflection notes. The first author also reviewed and summarised the changes made to the eligibility and recruitment process (e.g. ethics amendments) to document how the research team responded to the major recruitment challenges. Using an inductive approach to thematic analysis [22] author MF identified emergent subthemes within the data. Subthemes were checked and verified by authors TC and JG.

### Ethics approval

Ethics approval for the project was provided by the Human Research Ethics Committee (HREC) of the Townsville Hospital and Health Services District (HREC/15/QTHS/220), the HREC of the Northern Territory Department of Health and Menzies School of Health Research (HREC-2015-2491), James Cook University (Approval no. H6489) and the University of Queensland Behavioural and Social Sciences Ethical Review Committee (Approval no. 2015001591).

## Results

In total 62 patients were identified as being eligible for participation, with approximately 500 contact points recorded in the database between project staff and hospital staff, patients or their family members during the consent and recruitment process. Twenty-four patients were recruited to the study. Figure 1 illustrates the prominent recruitment challenges and the amendments implemented (Boxes 5–8): 1) Patients DAMA and ‘absconding’; 2) discharge prior to formal emergence from PTA as per the Westmead PTA Scale; 3) Patients under adult guardianship orders; 4) Narrow participant eligibility criteria and 5) Coordinating around patient comments and treatment. These challenges are described below. How the research team has also responded to these challenges is included.

### Discharging against medical advice and ‘absconding’

DAMA, also referred to as ‘self-discharge’, taking own leave or ‘absconding’ were the prominent recruitment challenges (Box 5). DAMA involves an admitted patient notifying hospital staff of their departure and occurs before discharge is advised by the treating practitioner, while absconding on the other hand, involves premature departure without notification. One in five patients (19.4%) identified as meeting eligibility criteria DAMA or ‘absconded’ from the hospital. With high rates of DAMA and absconding among Indigenous patients generally [23, 24], early departure from the hospital was the main reason eligible patients were not approached by research staff to participate in the project. It was common for patients to DAMA or abscond within hours of transfer from the Intensive Care Unit (ICU) to an acute ward, leaving limited opportunity for allied health staff to determine a patient’s consent capacity, for example: “…*our ICU gent was out on the [surgical] ward for about an hour before he self-discharged against medical advice.”* (email from allied health staff). In another example, it was recommended by an allied health contact that an eligible patient who had maxillofacial surgery following an assault not be approached until further into his recovery. The patient self-discharged three days post-surgery, without being approached by research staff.

Patients reported relationship issues, financial matters, difficulty sleeping in the hospital, belief that they did not have injury requiring hospitalisation and negative interaction with hospital staff as reasons for their desire to leave hospital (Box 8). These concerns also affected patients’ readiness to engage with research staff and consider project participation. In one example, a patient described feelings of distrust in hospital staff after the accusation that he had consumed alcohol while on approved hospital leave, … “*“hospital staff accused me of drinking on the weekend. Just because I’m black, I’ve been out drinking.*” *The patient stated that he no longer wants to stay at the hospital. He stated that he was happy to stay to get better but doesn’t want to stay if the staff do not believe him.”* These claims also impacted on caregivers’ and thoughts about what perceptions staff have of the family’s capacity to care for their family member: “*She [family member] reported that they [family member and patient] were angry because the doctors and nurses assume ‘we allowed him to be drinking over the weekend’.”*

Other patients were motivated to DAMA by the potential fraudulent use of their personal bank accounts:

“*[Patient] was agitated and was wanting to leave the ward to go to the bank. She was concerned that her money was going to be used by family and others.”*

*“[Caregiver] stated that some of her family have visited the patient and asked him for money. Concerns about his money and who has access to it have made the patient agitated and restless. [Caregiver] reported that she had arranged for the hospital to contact her to approve visitors while [patient name] was in hospital*.”

Similar to matters raised by patients in our project, Indigenous peoples’ experiences of hospital care generally can contain issues with hospital staff, loneliness [25], miscommunication regarding diagnosis, treatment and prevention [26] and disempowerment within healthcare systems [27]. Access to or dependency on alcohol can be another common reason patients desire to leave hospital [23, 28]. On occasions, family members of patients encouraged self-discharge:

*“some of the family members visiting [patient’s name] were heavy drinkers and would ask [patient’s name] to come and drink with them.”*

‘Safety Specialists’ or ‘Personal Care Assistants’ were assigned for one-on-one supervision of some eligible patients who were determined as high-risk of DAMA or due to concerns including risk of falling or other harm to self. The use of this one-on-one supervision does not necessarily prevent DAMA due to restrictions on the use of physical restraint without the appropriate legal processes being undertaken.

As described in Fig. 1 (Box 5), project and hospital staff identified that earlier contact with eligible patients was required to improve recruitment. When research staff were notified of an eligible patient, they collected anecdotal information regarding the patient’s likelihood of self-discharge, and efforts were made to meet with patients as quickly as possible. If research staff were unable to visit the patient shortly after notification, an allied health worker attached to the study yet not involved in the patient’s care spoke to the patient about the project. Arrangements were then made for research staff to speak with interested patients by telephone. The original process (Box 4) was also altered to allow contact with a caregiver earlier. These changes have reduced the number of eligible patients not approached to participate due to DAMA or ‘absconding’.

### Clinicians are the best measure for determining suitability for consent

A significant challenge impeding recruitment was the process for determining a patient’s capacity to consent to participation (Box 6). Based on the original recruitment process (Box 3), patients were eligible to provide informed consent when it was determined they had emerged from PTA. While the Westmead PTA Scale was not specifically referred to in the original study protocol [5], it was standard practice for allied health staff to use the Westmead PTA Scale in accordance with its protocol to determine PTA status for many patients post head injury. As described in Box 3, the Westmead PTA Scale’s protocol determines that an individual has emerged from PTA once they score all twelve questions correctly for three days consecutively. It was identified early in recruitment that patients’ performance on this assessment was not always considered by treating teams when determining discharge suitability. When PTA was considered, in certain cases a single score of 12/12 on the Westmead PTA Scale was deemed sufficient evidence by their treating medical officer that a patient had emerged from PTA and was suitable for discharge. The varying conditions under which PTA and discharge suitability are determined resulted in some eligible patients being discharged prior to being identified as eligible for the study.

Patients under interim adult guardianship orders (9.6% of patients identified as meeting the eligibility criteria) were impacted most by this issue. During recruitment, no patients under an adult guardianship order emerged from PTA. On occasion, allied health staff liaised with medical officers to determine if some patients under guardianship orders had the ability to understand the requirements of participating in the project and the ability to consent despite not performing well on the Westmead PTA Scale. Other concerns raised include the scale’s limited validity for use with Indigenous Australians and patients’ pre-injury ability to pass the scale.

In response, consent capacity was now determined by the liaising verbally with the treating medical team, rather than emergence from PTA (Box 6) as per the standardised protocol. As in the original recruitment process, the nominated caregiver was able to assist the research team to gain consensus about a patient’s capacity.

### Coordinating around patient commitments and treatment

The importance of building rapport is well documented in the literature regarding Indigenous research. In order to have adequate time to discuss the project and build rapport with an eligible patient, it was common for research staff to spend time with a patient without commencing data collection. These rapport-building meetings lasted approximately 30 to 45 min.

To ensure data collection did not interfere with patient treatment and discharge planning, the research team attempted to navigate around appointments and other commitments including family visits, finalising any paperwork required (for example medical certificates) and completing pension payment paperwork. In the following example, the project staff member travelled to the hospital at a time suitable to the Indigenous liaison officer (ILO) so the ILO could introduce the project staff member to the patient. Upon arrival, the ILO advised the project staff member he had to assist the patient with urgent banking matters prior to speaking with the project staff member: *“….he (ILO) has been working with the patient and is assisting with the patient getting access to his bank account and money. He stated that the patient has lost his bank cards. [ILO] and [project staff member] arranged to meet with the patient in 30 minutes.”*

Although efforts were made by allied health staff to coordinate a suitable time for contact with the patient, it was common for research staff to re-schedule further hospital visits. Patient appointments with doctors and hospital staff were commonly completed on an impromptu basis. Moreover, police officers also presented to the hospital to visit patients in circumstances where the mechanism of injury required further investigation. Such appointments left patients feeling understandably fatigued. To ensure data collection did not obstruct other commitments, outstanding data collection was completed shortly after patients were discharged.

Overall, research staff contact with both patient and hospital staff to identify, consent and recruit each patient ranged from 3 to 15 contacts (phone calls and hospital visits). On average, research staff spent approximately 5.25 h (range 3.25–11.5 h) to recruit each patient and approximately 2 h (range 0.25–5.25 h) hours with each non-recruited patient.

### Patients under adult guardianship orders

In the original recruitment process, caregivers were requested to sign a consent form permitting a patient under an adult guardianship order to participate in the project. The completion of this process relied on the caregiver being present at the hospital. Where the Public Guardian was the nominated guardian, awaiting approval could delay research staff completing an assessment with a patient. Liaison with the Office of the Public Guardian was imperative to managing this challenge.

### Criminal justice involvement

Although an uncommon barrier, some eligible patients were in the custody of corrective services at the time of hospital admission: “*The patient had two QCS* [Queensland Corrective Services] *officers guarding him. One of the officers stated that although the patient has been flagged as suitable, I did not have the correct approvals to speak with him given he was in custody*.” To address this issue, approval was sought from corrective services to access patients on an individual basis. Unfortunately, patient discharge from the hospital to a correctional facility occurred prior to approval, preventing any patient contact with research staff.

### Increasing access to eligible patients through additional recruitment sites

The original study protocol included two recruitment sites, RDH and TTH (Box 1). During the project consultation period, the Chief Investigators received guidance that almost all patients who sustain a TBI from Northern- and Western Queensland would be admitted to TTH as it is a tertiary referral centre for neurosurgical services. Further discussions with TTH staff during the earlier stages of recruitment indicated that patients who sustain a milder head injury may receive care from the Cairns or Mount Isa hospitals. These two hospitals were thus added as recruitment sites to engage a larger pool of patients from these catchment areas (Box 7).

### Broadening the participant eligibility criteria

The original eligibility criteria (Box1) was suggested to be too narrow, confining recruitment to certain wards. Patients with a loss of consciousness may stay in the Emergency Departments (ED) or units attached to the EDs for less than 24-h and not require admission to a ward (Box 7). As these patients were not admitted to acute wards, the existing recruitment strategy with our allied health contacts would not identify them. To support the ethics amendment submission for the removal of the > 24 h admission criteria and develop recruitment processes within two of the four hospitals’ ED extended management units, consultation across multiple departments and letters of support were required.

### Recruitment from community health services

To overcome the challenges experiences in hospital recruitment, the research team engaged with community-based Indigenous health and rehabilitation services to recruit community members who have experienced a TBI. To participate in the project, community members were still required to have attended hospital for treatment within the last 6 months and met all other eligibility criteria (outlined in Fig. 1). Recruitment practices were tailored according to what was appropriate for service staff and their clients. Strategies included posters displayed in the health services with information on the project and treating clinicians and health workers speaking to patients of the health service who met the eligibility criteria. Interested patients could self-refer themselves to the project by contacting project staff to participate or provide consent to allow the clinician to provide the research team with the person’s contact details. Although this recruitment strategy prevented survey data collection at the first-time point (prior to hospital discharge), qualitative information about the hospital and transition period could still be provided by patients during the subsequent time points (three and six months after discharge).

## Discussion and recommendations

The primary aim of this study was to identify the major challenges preventing engagement and recruitment of Indigenous Australian patients admitted for TBI to a longitudinal project. DAMA and ‘absconding’ and patients remaining in PTA as per the Westmead PTA Scale were the major challenges identified. Determination of capacity and emergence from PTA is more amendable to modification, for example through using medical practitioners to determine capacity rather than the Westmead PTA Scale, in comparison to DAMA and ‘absconding’.

Considering the hospital admission rates of TBI of Indigenous Australians [1, 2], the research team received a lower number of identified suitable patients during recruitment. The circumstances in which Indigenous Australians present to hospital for a TBI and the experiences they report while in hospital may have contributed to challenges in identifying them for the project. Compared to non-Indigenous Australians, Indigenous Australians are more likely to present to hospital with a traumatic brain injury following an assault [2]. Falls are the main cause of TBI among non-Indigenous Australians [2]. Following an assault, Indigenous patients may have a range of needs that require addressing following their hospital admission including access to housing, child-related responsibilities and assisting with police investigations surrounding the assault [29]. While in hospital, Indigenous patients report feelings of loneliness, difficulties engaging with treating clinician and feeling excluded from discussion relating to their health care and treatment [29]. The needs and experiences of Indigenous TBI patients while in hospital may mean some patients do not come to the attention of clinicians for the project. Patient access to treatment is paramount and research recruiting through hospitals should ensure they do not impinge upon patients’ rights to health care.

The challenge of recruiting patients prior to DAMA and ‘absconding’ in this project is reflective of the discharge process many Indigenous patients admitted to hospital in Australia experience [2]. Indigenous Australians admitted for TBI discharge themselves from hospital at a rate four times higher than non-Indigenous Australians (one in 10 Indigenous patients with TBI compared to one in forty non-Indigenous patients) [2]. While efforts were made to improve access by project staff to patients within the hospital environment, DAMA remained an ongoing challenge to manage. The project acknowledged the importance of capturing the transition experience of patients who DAMA, as this may not be similar to patients who discharge through proper channels. This was addressed through implementing a community-based recruitment strategy. Patients who discharge themselves from hospital are shown to go onto experience poorer outcomes [30, 31]. Greater understanding of the transition experiences for Indigenous patients with TBI who DAMA is imperative to improving how hospital and community-based services respond to them and their needs [2].

Akin to other studies recruiting ‘hard to reach’ populations [14], adjustable project timelines and budgets were necessary to ensure appropriate responses could be made to this challenge. Our reflections also demonstrate that a carefully crafted yet flexible recruitment protocol is important to maximise recruitment. Making modifications to the original recruitment protocol (Boxes 1–4) was time intensive, requiring engagement with new hospital contacts, the procurement of written support, submission of ethics amendments to the relevant committees and trialing of new processes.

Sustainable partnerships between research- and hospital staff were paramount to recruitment. Unlike community-based projects where researchers have greater control and involvement in recruitment [32–34], the research team were reliant on hospital staff identifying eligible patients. It should be recognised that the task of identifying eligible patients for the project was accepted by hospital staff who already held full caseloads. To develop mutual expectations and referral pathways which are able to be embedded into routine practice, the research relationship must be afforded time to be negotiated between all parties. To ensure researchers are able to pursue regular updates regarding patients and account for staff absence, it is also pertinent to have multiple contacts within and across wards. We also recommend the use of a centralised recruitment officer (see recommendation point one).

In turn, we make a number of recommendations which can guide future studies:

1. *Ability to employ a hospital staff member as a centralized recruiter at each hospital site.* As used in rehabilitation environments elsewhere [35], a centralized recruiter would act as a liaison between the research team and potential research participants. Based at on hospital grounds, a centralized recruiter could minimise many of the described delays in the identification, consent and data collection process. A centralised recruiter may be able to more readily access health system data and work across wards. Supporting research projects operating in the hospital would be the recruiter’s primary task and would therefore not be directly part of a patient’s treating team. Remove any potential complexities surrounding clinicians and allied health staff being required to report information to authorities they may receive in a researcher role.
2. *Proposed recruitment processes should include specific detail on criteria for participation.* Unnecessary criteria or description of recruitment processes in a study protocol may result in recruitment processes being unable to be implemented in some wards. For example, the > 24 h admission criteria reduced the wards the project could implement recruitment processes. To make modifications to recruitment processes and patient eligibility criteria, the research team consulted the ethics committee and submitted ethics amendments in line with research guidelines [36];
3. *Engagement with patients, consent and data collection processes should be flexible*. It is likely that in this environment, the completion of consent and data collection may require a researcher to meet with a patient on multiple occasions over multiple days. Consent and data collection processes need to include additional time to account for patient treatment, meeting with clinicians and hospital staff, other non-health needs (e.g accommodation needs), other meetings (e.g. contact with police), discharge planning, arrangement of interpreters, patient health (e.g fatigue, loneliness) and patient travel arrangements. In some circumstances, data collection may not be possible in the hospital.
4. *Indigenous staff involvement at recruitment and data collection is essential* including Indigenous research officers, ILOs and interpreters. This will ensure culturally safety of Indigenous patients.
5. *Ensure early liaison with non-hospital services that are likely to have involvement with patients in the hospital* including the Alcohol and Drug Services and the Office of the Public Guardian;
6. *The research team should regularly reflect on their recruitment experiences* to identify challenges early and respond appropriately;

In addition to identifying the challenges of recruiting patients, the findings also identified several examples of patients’ describing poor interactions with hospital staff. As reported here and elsewhere, poor communication with treating clinicians can contribute towards desire to self-discharge [28] as well as patients’ limited understanding of their injury and their understanding of the importance of completing treatment [37, 38]. Most hospitals, including the two in this study, already have a requirement for staff to complete ‘cultural competency’ training. A review of existing training packages to address obvious shortfalls in the cultural competency skill level of some hospital employees is recommended. Finally, it is recommended better practices to obtain regular feedback from Indigenous patients about the performance of hospital staff in meeting their needs are implemented.

## Conclusion

Hospital-based research studies involving Indigenous patients present unique barriers to recruitment that must be explicitly addressed to ensure projects maximize recruitment and are thus able to give voice to this unique, underserved population. While this process identified key processes for hospital recruitment, these remain our own reflections in recruiting Indigenous Australian patients following a TBI. It is essential for researchers investigating other health outcomes to report their experiences of recruiting Indigenous patients from the acute and rehabilitation environment. This will lead to greater guidance to support current and future researchers to work in a culturally responsive manner with Indigenous patients and their families, and to develop informed processes that can anticipate potential recruitment challenges.

## Data Availability

Not applicable.

## Abbreviations

DAMA: Discharging against medical advice
ED: Emergency Department
HREC: Human Research Ethics Committee
ICU: Intensive Care Unit
NHMRC: National Health and Medical Research Council
PTA: Post-traumatic amnesia
QCS: Queensland Corrective Services
RDH: Royal Darwin Hospital
TBI: Traumatic brain injury
TTH: The Townsville Hospital
